# Activity of Delafloxacin and Levofloxacin against Stenotrophomonas maltophilia at Simulated Plasma and Intrapulmonary pH Values

**DOI:** 10.1128/spectrum.02705-21

**Published:** 2022-07-11

**Authors:** Alesia Vialichka, Mark Biagi, Kevin Meyer, Tiffany Wu, Aisha Shajee, Xing Tan, Eric Wenzler

**Affiliations:** a College of Pharmacy, University of Illinois Chicago, Chicago, Illinois, USA; b College of Pharmacy, University of Illinois Chicago, Rockford, Illinois, USA; Hartford Hospital

**Keywords:** susceptibility testing, time-kill assay, fluoroquinolone, delafloxacin, levofloxacin, pharmacodynamics, pH, acidic environment, epithelial lining fluid, pneumonia, *Stenotrophomonas maltophilia*

## Abstract

Fluoroquinolones have become a popular treatment option for Stenotrophomonas maltophilia infections. Although levofloxacin is most commonly used, delafloxacin demonstrates comparable *in vitro* activity when evaluated under standard susceptibility testing conditions at neutral pH. At acidic pH, the activity of the anionic delafloxacin is improved, while the activity of the zwitterionic levofloxacin is reduced. Because the human respiratory tract has a pH of ~6.6 and is the most common site of S. maltophilia infection, it is vital to understand the activity of these agents in this environment. Therefore, levofloxacin and delafloxacin were tested against clinical S. maltophilia isolates via broth microdilution testing (*n* = 37) and time-kill analysis (*n* = 5) in neutral cation-adjusted Mueller-Hinton broth (CAMHB) (pH 7.3) and acidic CAMHB (aCAMHB) (pH 6.5). In CAMHB, MIC_50_ values were similar between levofloxacin and delafloxacin (8 mg/L versus 8 mg/L). In aCAMHB, levofloxacin MICs did not change, while delafloxacin MICs decreased by a median of 4 log_2_ dilutions (MIC_50_ values of 8 mg/L versus 0.25 mg/L). In time-kill analyses, levofloxacin and delafloxacin at the maximum drug concentration for the free drug (*fC*_max_) were bactericidal against 3 and 2 isolates in CAMHB, respectively. In aCAMHB, levofloxacin was not bactericidal against any isolate, while delafloxacin was bactericidal against the same 2 isolates. Relative to CAMHB, levofloxacin activity was reduced by 2.5 log_10_ CFU/mL in aCAMHB, whereas delafloxacin activity was increased 2.7 log_10_ CFU/mL. Although the bactericidal activity of levofloxacin against S. maltophilia was attenuated in an acidic environment in this study, the increased potency of delafloxacin at pH 6.5 did not translate into improved bactericidal activity in time-kill analyses, compared to pH 7.3.

**IMPORTANCE** Stenotrophomonas maltophilia most often infects the lungs, where the physiologic environment is naturally slightly acidic (pH ~6.6), compared to most parts of the body (such as the bloodstream), which have neutral pH values (~7.4). Pneumonia due to S. maltophilia is often treated with the antibiotic levofloxacin, despite the activity of levofloxacin being known to be impaired at acidic pH. Unfortunately, currently available methods for susceptibility testing of levofloxacin against S. maltophilia are performed at a neutral pH and therefore may not accurately represent the activity of levofloxacin at the site of infection in the lungs. A similar but newer antibiotic in the same class as levofloxacin, namely, delafloxacin, is not affected by being in an acidic environment and may actually work better at lower pH values. Therefore, the purpose of this study was to investigate whether one drug might be better than the other in this setting by testing each agent’s ability to kill S. maltophilia at pH 7.3 and pH 6.5. These findings could then be used to design confirmatory studies that may ultimately impact which drug is given to patients with lung infections due to S. maltophilia.

## INTRODUCTION

Stenotrophomonas maltophilia is one of the most commonly encountered carbapenem-resistant organisms in the hospital setting but has far fewer viable treatment options, compared to carbapenem-resistant *Enterobacterales* and Pseudomonas aeruginosa (1). The intrinsic dual β-lactamases of S. maltophilia effectively eliminate most typical first-line treatment options with high efficacy and low toxicity (e.g., β-lactams), leaving sulfamethoxazole-trimethoprim (SMX-TMP) as the traditional drug of choice, given its reliable *in vitro* susceptibility (2, 3). Due primarily to tolerability and toxicity issues associated with SMX-TMP, there has been a renewed interest in exploring alternative treatment options, especially the fluoroquinolones (FQs) (4). Contemporary data demonstrate more reliable susceptibility testing results, superior *in vitro* pharmacodynamics, and improved antibiofilm activity of the FQs, compared to SMX-TMP, while available retrospective clinical outcome data are comparable (5–12). Together, these factors have driven U.S. clinicians to prescribe FQs almost twice as often as SMX-TMP for definitive therapy of serious S. maltophilia infections, such as bacteremia and lower respiratory tract infections (1, 13).

Within the FQ class, the availability of Clinical and Laboratory Standards Institute (CLSI) breakpoints for levofloxacin against S. maltophilia drives much of its clinical use and investigation into its antibacterial activity (14). Despite this, other FQs, such as ciprofloxacin, delafloxacin, and moxifloxacin, have demonstrated similar or better *in vitro* potency, compared with that of levofloxacin, against S. maltophilia (15–18). Delafloxacin is a novel anionic FQ that has displayed improved *in vitro* activity, compared with levofloxacin, against common Gram-positive and Gram-negative pathogens under standard testing conditions (19). Uniquely, when tested at acidic pH values (≤5.5), the intracellular accumulation and relative potency of the weakly acidic delafloxacin can increase as much as 10-fold, relative to neutral pH (20). Conversely, the antibacterial activity of the traditional zwitterionic FQs is reduced by as much as 32-fold under acidic conditions, such as those present in the human urinary and respiratory tracts (21). Because the most common and challenging site of S. maltophilia infection is the respiratory tract, which is known to have an epithelial lining fluid (ELF) pH of ~6.6, delafloxacin may have an advantage over other FQs in this environment (3, 22, 23). This is especially critical in the setting of pneumonia, which further decreases intrapulmonary pH, impairing innate host defenses and impeding the antibacterial activity of FQs by slowing bacterial growth and inducing biofilm formation (24, 25). Importantly, the potential advantage of delafloxacin would not be appreciated by routine antimicrobial susceptibility testing performed in media with a neutral pH (~7.4), as is currently recommended. Therefore, the objective of this study was to evaluate and compare the *in vitro* activities of delafloxacin and levofloxacin via broth microdilution (BMD) testing and time-kill analyses in simulated plasma and ELF acid-base environments against a collection of challenging clinical S. maltophilia isolates.

(This work was presented in part as a poster at the 2018 ASM Microbe meeting, San Francisco, CA.)

## RESULTS

**FIG 1:**
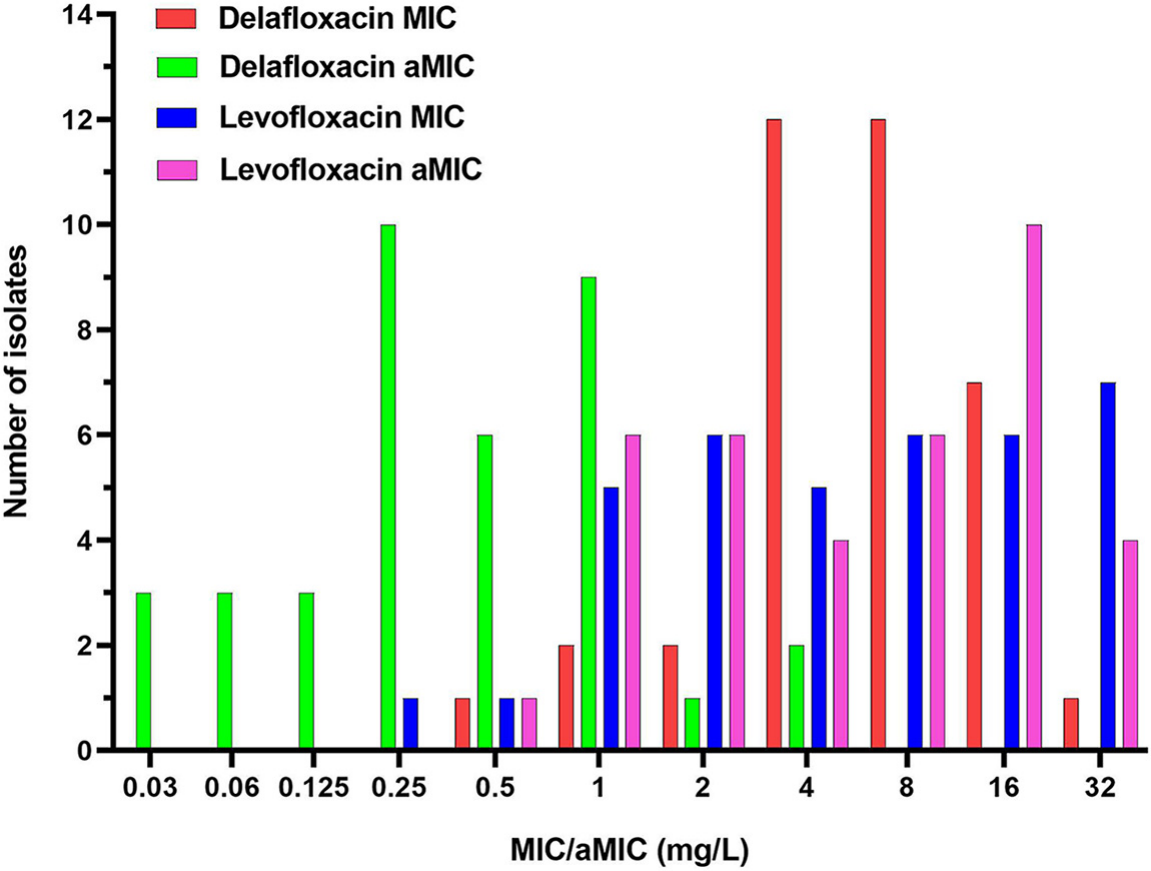
MIC and aMIC distributions for delafloxacin and levofloxacin when tested in CAMHB (pH 7.3 ± 0.2) and aCAMHB (pH 6.5 ± 0.2) against 37 clinical S. maltophilia isolates.

The MIC_50_, acidic MIC_50_ (aMIC_50_), MIC_90_, aMIC_90_, MIC range, aMIC range, and susceptible proportions for delafloxacin and levofloxacin against all 37 S. maltophilia isolates in cation-adjusted Mueller-Hinton broth (CAMHB) and acidic CAMHB (aCAMHB) are presented in Table 1. No significant differences in MIC or aMIC values were observed when stratified by acquisition setting, culture source, or geographic location (data not shown). When tested in CAMHB, MIC_50_ and MIC_90_ values appeared similar for delafloxacin and levofloxacin, although just 1 isolate was susceptible (MIC of ≤0.5 mg/L) to delafloxacin (2.7%) versus 13 (35.1%) to levofloxacin. As displayed in Fig. 1, 65% of the 37 isolates had a delafloxacin MIC of 4 or 8 mg/L in CAMHB, compared to just 30% against levofloxacin. In aCAMHB, delafloxacin aMICs decreased by a median of 4 log_2_ dilutions (range, 1 to 6 log_2_ dilutions), compared to those in CAMHB, and susceptibility increased to 67.6%, while no significant change was observed in levofloxacin MICs or susceptibility (Fig. 1 and Table 1).

**TABLE 1:** Activity of delafloxacin and levofloxacin in CAMHB (pH 7.3 ± 0.2) and aCAMHB (pH 6.5 ± 0.2) against 37 clinical S. maltophilia isolates

Medium and drug	MIC (mg/L)			aMIC (mg/L)			% susceptible^*a*^
	MIC_50_	MIC_90_	Range	aMIC_50_	aMIC_90_	Range	
CAMHB							
Delafloxacin	8	16	0.5 to 32				2.7
Levofloxacin	8	≥32	0.25 to ≥32				35.1
aCAMHB							
Delafloxacin				0.25	1	0.03 to ≥4	67.6
Levofloxacin				8	32	0.5 to 32	35.1

a Susceptibility interpretations are based on CLSI interpretive criteria for levofloxacin against S. maltophilia (MIC of ≤2 mg/L) and FDA interpretive criteria for delafloxacin against P. aeruginosa (MIC of ≤0.5 mg/L).

The individual MIC and aMIC values of each agent in neutral and acidic media for the 5 S. maltophilia isolates selected for time-kill experiments are shown in Table 2, along with the purported maximum drug concentration (*C*_max_) and the area under the concentration-time curve for the free drug (*f*AUC), in relation to the MIC/aMIC for each. One isolate was susceptible to delafloxacin, 1 was intermediate, and 3 were resistant in CAMHB, whereas 4 were susceptible and 1 was resistant in aCAMHB. Three isolates were levofloxacin susceptible and 2 were resistant regardless of the test medium, making overall susceptibility 50% for delafloxacin and 60% for levofloxacin across the 10 triplicate BMD tests performed against isolates included in time-kill analyses (5 in CAMHB and 5 in aCAMHB). The delafloxacin *C*_max_/MIC and *f*AUC/MIC values were above the targets of 12.2 and 60, respectively, only for the isolate with the lowest MIC (STMA-1, with a MIC of 0.5 mg/L), while both indices were well above the targets for 4 of 5 isolates in aCAMHB. While levofloxacin *C*_max_/MIC values were adequate only for the 2 isolates with the lowest MICs (STMA-1 and STMA-2) and not for any isolate according to the aMIC, the predicted *f*AUC to MIC/aMIC threshold was achieved for STMA-1, STMA-2, and STMA-4 (Table 2).

**TABLE 2:** MICs, *C*_max_/MIC, and *f*AUC/MIC ratios of delafloxacin and levofloxacin in CAMHB (pH 7.3 ± 0.2) and aCAMHB (pH 6.5 ± 0.2) against 5 S. maltophilia isolates included in time-kill experiments

Isolate	Delafloxacin^*a*^						Levofloxacin^*b*^					
	MIC (mg/L)	*C*_max_/MIC	*f*AUC/MIC	aMIC	*C*_max_/aMIC	*f*AUC/aMIC	MIC (mg/L)	*C*_max_/MIC	*f*AUC/MIC	aMIC	*C*_max_/aMIC	*f*AUC/aMIC
STMA-1	0.5	17.9^*c*^	72^*c*^	0.03	298^*c*^	1200^*c*^	0.25	37.2^*c*^	624^*c*^	1	9.3	156^*c*^
STMA-2	1	8.9	36	0.125	71.5^*c*^	288^*c*^	0.5	18.6^*c*^	312^*c*^	1	9.3	156^*c*^
STMA-3	4	2.2	9	0.5	17.9^*c*^	72^*c*^	8	1.2	19.5	8	1.2	19.5
STMA-4	8	1.1	4.5	0.25	35.8^*c*^	144^*c*^	1	9.3	156^*c*^	1	9.3	156^*c*^
STMA-5	32	0.3	1.1	≥4	2.2	9	≥32	0.3	4.9	32	0.3	4.9

a *C*_max_ of 8.94 mg/L after a single 300- mg dose, with an estimated *f*AUC of 36 mg · h/L.

b *C*_max_ of 9.3 mg/L after a single 750-mg dose, with an estimated *f*AUC of 156 mg · h/L.

c *C*_max_/MIC or *C*_max_/aMIC above the target of 12.2 and/or *f*AUC/MIC or *f*AUC/aMIC of at least 60 for delafloxacin and 55 for levofloxacin.

**FIG 2:**
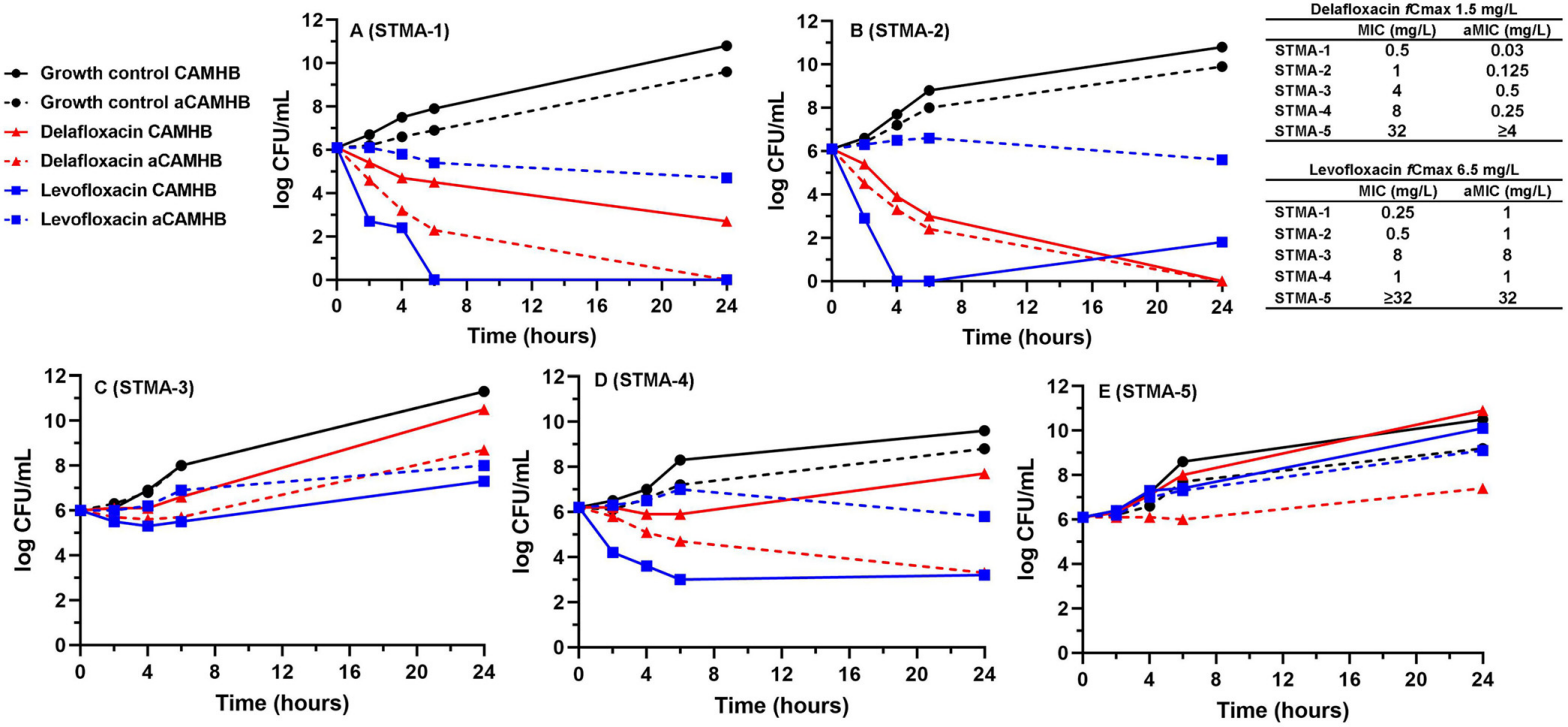
Mean log_10_ CFU per milliliter versus time profiles for delafloxacin (red triangles) and levofloxacin (blue squares) at *fC*_max_ against 5 S. maltophilia strains (A to E). Experiments performed in CAMHB (pH 7.3 ± 0.2) are shown as solid lines, and experiments performed in aCAMHB (pH 6.5 ± 0.2) are shown as dashed lines. Curves represent average concentrations from triplicate experiments.

Mean bacterial concentration (CFU per milliliter) versus time profiles for delafloxacin and levofloxacin at the *C*_max_ for the free drug (*fC*_max_) against each of the 5 S. maltophilia isolates in CAMHB and aCAMHB are shown in Fig. 2. Variability in bacterial concentrations was negligible across the 60 active drug time-kill experiments performed (mean intraassay and interassay coefficients of variation [CVs] of 2.6% and 1.4%, respectively). The bacterial concentrations at 24 h in the drug-free control wells for STMA-1, STMA-2, and STMA-5 were >1 log_10_ CFU/mL lower in aCAMHB, compared to those in CAMHB, consistent with the known poor tolerance of S. maltophilia for acidic environments (Fig. 2) (25). The bactericidal activity of delafloxacin in time-kill experiments correlated moderately well with the MICs obtained and the respective *C*_max_/MIC and *f*AUC/MIC ratios but not the aMICs (Table 2). In CAMHB, delafloxacin was bactericidal only against the 2 isolates with MICs below its *fC*_max_ of 1.5 mg/L (Fig. 2A and B). Levofloxacin *f*AUC better correlated with bactericidal activity than did *C*_max_ against MIC/aMICs. In CAMHB, levofloxacin was bactericidal against the 3 susceptible isolates with MICs below its *fC*_max_ of 6.5 mg/L and above the *f*AUC/MIC target of 55 (Fig. 2A, B, and D). The median change at 24 h from time 0 h was 1.5 log_10_ CFU/mL (range, −6.1 to 4.7 log_10_ CFU/mL) for delafloxacin and −2.9 log_10_ CFU/mL (range, −6.1 to 4 log_10_ CFU/mL) for levofloxacin in CAMHB. Although aCAMHB reduced the delafloxacin aMIC to below its *fC*_max_ and increased its *C*_max_/aMIC and *f*AUC/aMIC ratios to >12.2 and >60, respectively, in 4 of 5 strains, bactericidal activity in time-kill analyses was achieved only against the same 2 isolates as in CAMHB (STMA-1 and STMA-2) (Fig. 2A and B), although a nearly bactericidal 2.8-log_10_-unit kill was achieved against STMA-4 (Fig. 2D). Interestingly, although 3 isolates maintained susceptible aMICs less than the levofloxacin *fC*_max_ and *f*AUC/aMIC values of >55, bactericidal activity was not achieved against any strain in aCAMHB. The median changes at 24 h were −2.8 log_10_ CFU/mL (range, −6.1 to −2.7 log_10_ CFU/mL) for delafloxacin and −0.4 log_10_ CFU/mL (range, −1.5 to −3 log_10_ CFU/mL) for levofloxacin in aCAMHB. Examining the agents individually revealed that, compared to CAMHB, testing delafloxacin in aCAMHB resulted in an additional 2.7-log_10_-unit reduction (range, 0- to 4.3-log_10_-unit reduction) in the bacterial concentration at 24 h, whereas it resulted in a 2.5-log_10_-unit increase (range, 1- to 4.6-log_10_-unit increase) at 24 h for levofloxacin. Finally, visual inspection of Fig. 2 demonstrates that the acidic pH of aCAMHB resulted in qualitatively better activity, compared with CAMHB, for delafloxacin in 4 of 5 strains and qualitatively worse activity for levofloxacin in 4 of 5 strains.

## DISCUSSION

S. maltophilia is an extremely challenging pathogen with very limited treatment options due to its prolific intrinsic resistome (26, 27). Although SMX-TMP has traditionally been considered the drug of choice, there are no rigorous clinical data to support its use, and the optimal therapy remains unknown (13). Meanwhile, clinicians in U.S. hospitals continue to look for alternatives to SMX-TMP for S. maltophilia and are supported by emerging large-scale comparative effectiveness data suggesting that treatment with levofloxacin may be associated with reduced mortality rates, particularly among patients with pneumonia (13). Levofloxacin is the most commonly utilized FQ for S. maltophilia, owing primarily to the availability of CLSI breakpoints, although other FQs, including delafloxacin, possess adequate *in vitro* activity. Delafloxacin is unique among the FQs in that it is the only nonzwitterionic molecule and thus its potency is increased in acidic environments, whereas the activity of the traditional FQs is diminished at low pH. Because S. maltophilia most commonly infects the respiratory tract, which is known to have an acidic pH (5.6 to 6.7), especially during infection, it is vital to understand the expected activity of antimicrobial treatment options in this environment (28). This is particularly true when the environment in which *in vitro* susceptibility testing is performed does not accurately represent the physiologic conditions at the site of infection (29, 30).

The primary findings from the current work are 2-fold. First, the presence of an acidic environment appeared to adversely impact the antibacterial activity of levofloxacin more than it benefitted delafloxacin. Although the bactericidal activity of delafloxacin was predicted to improve after aMICs decreased and *C*_max_/aMIC and *f*AUC/aMIC ratios correspondingly increased, it did not improve, and the same level of activity was maintained as was observed at a neutral pH. Conversely, the bactericidal activity of levofloxacin observed in CAMHB (3 of 5 isolates) was abolished when tested in aCAMHB via time-kill analysis, despite adequate predicted *f*AUC/aMIC. Overall, the acidic environment of the aCAMHB improved the activity of delafloxacin by a median of 2.7 log_10_ CFU/mL over CAMHB, while it worsened the activity of levofloxacin by a median of 2.5 log_10_ CFU/mL. While these results do not strongly support delafloxacin as an alternative agent, they do question the adequacy of levofloxacin’s antibacterial activity against S. maltophilia at target infection sites. To our knowledge, the only other study evaluating the impact of pH changes on the activity of delafloxacin against Gram-negative pathogens included 9 Escherichia coli and 7 Klebsiella pneumoniae strains with high-level ciprofloxacin resistance (31). The authors tested MICs for delafloxacin and ciprofloxacin via BMD testing at neutral pH and in urine samples from 100 patients with pH values ranging from 5 to 8.3. In contrast to our findings, the median change in the delafloxacin MIC was only 1 log_2_ dilution at pH ≤6, and there was no change at pH 6.1 to 7.0. Ciprofloxacin MICs remained the same or changed by only 1 log_2_ dilution as well. While these results also suggest that there is no advantage of delafloxacin over other FQs at acidic pH, they may indicate that the impact of pH on the activity of the FQs is species specific.

Second, our study adds to the existing body of evidence demonstrating consistent and significant discordance between *in vitro* MICs and the antibacterial activity of antimicrobial agents against S. maltophilia. In particular, the inability to achieve bactericidal activity with monotherapy *in vitro* despite attainable MIC/aMIC values, as demonstrated again here, is disconcerting but commonplace with this pathogen (11, 12, 32, 33). This disparity is often attributed to the low growth rate and high mutation frequency of S. maltophilia and has been observed across every class of drugs active against this pathogen (34). The magnitudes of the increase in aMIC for levofloxacin and the decrease in aMIC for delafloxacin in an acidic environment in our study are consistent with previous data evaluating the intracellular and extracellular activity of FQs against Staphylococcus aureus (35). Similar to our work, the authors demonstrated that, despite the observed shift in MIC value, the minimum bactericidal concentrations (MBCs) correlated better with the MICs measured at pH 7.3 versus pH 5.0. They also observed poor correlation between antibacterial activity observed in time-kill analyses and MBC/MIC ratios. While they noted that the impact of pH on the activity of FQs is much more pronounced among Gram-positive pathogens, they also established that the etiology of the improved activity of delafloxacin in an acidic environment is not well understood and is likely multifactorial but is not related directly to improved interaction with the FQ target site or increased intracellular FQ concentrations. Although we did not observe the development of resistance in this study, previous work has also demonstrated that acidic conditions trigger S. maltophilia to upregulate expression of efflux pumps such as CmeB from the resistance-nodulation-cell division (RND) family, which has been implicated in high-level FQ resistance (36). These uncertainties and inconsistencies question the reliability of our current *in vitro* susceptibility testing methods to accurately represent antibacterial activity at the site of infection and further challenge our ability to optimize therapy against S. maltophilia.

While they are useful for evaluating drug-pathogen interactions in simulated human physiologic environments *ex vivo* (37), nonclinical *in vitro* methods such as the time-kill analyses utilized here are not without limitations. Although dynamic models are always preferred, the static nature of the time-kill experiments was adequate for this work, because each drug served as its own comparator for its activity in CAMHB versus aCAMHB, in order to determine whether one agent might be optimal in such an environment. The isolates included in this work were purposefully selected for their nonsusceptibility to SMX-TMP and/or levofloxacin, in order to (i) mimic a potential scenario in which a second-line agent like delafloxacin might be used, (ii) incorporate isolates with elevated FQ MICs to ensure that enough aMICs remained evaluable after the aCAMHB exerted its inverse effect on the MICs of the two agents, and (iii) allow for comparisons of antibacterial activity between agents and media while holding the drug exposure and the isolate constant. Although only 5 isolates were selected for time-kill analyses, each had 4 MIC/aMIC values across the two agents and two media tested, increasing the total number of MICs to 20, with 8 unique values ranging from 0.03 to ≥32 mg/L. Altering only the isolate’s MIC reduced the interstrain variability typically observed in time-kill studies that include a larger number of heterogeneous isolates in order to fill the MIC distribution and permitted direct comparisons of the antibacterial activity between drugs and media. Despite these study design strengths, time-kill analyses are inherently precursory; therefore, the findings from this work should be used to inform the design of future studies using dynamic pharmacokinetic/pharmacodynamic systems and incorporating more isolates and those with similar phenotypes.

In summary, despite divergent MIC changes when tested in an acidic environment, these changes did not seem to translate into improved bactericidal activity for delafloxacin over levofloxacin in this study. Although the antibacterial activity of levofloxacin was attenuated in an acidic environment, the activity of delafloxacin was not substantially improved in aCAMHB, compared to CAMHB. These data suggest that levofloxacin remains the FQ of choice for S. maltophilia, while additional studies are needed to determine the optimal therapeutic strategy for S. maltophilia infections.

## MATERIALS AND METHODS

### Bacteria and susceptibility testing.

A panel of 37 clinical S. maltophilia isolates that were not susceptible to either levofloxacin, SMX-TMP, or both, which had been collected through the SENTRY Antimicrobial Surveillance Program from 2017 to 2018, were included in all experiments. Species identification was confirmed at JMI Laboratories (North Liberty, IA) by standard biochemical tests and matrix-assisted laser desorption ionization–time of flight mass spectrometry (MALDI-TOF MS) (Bruker Daltonics, Billerica, MA). Isolates included community-acquired and nosocomially acquired strains collected primarily from the respiratory tract (*N* = 24, including 10 sputum, 8 tracheal aspirate, and 6 bronchoalveolar lavage fluid samples) across multiple continents. All isolates were maintained at −80°C in CAMHB (Teknova, Hollister, CA) with 20% glycerol and were subcultured twice on tryptic soy agar plates with 5% sheep blood prior to use.

Analytical-grade delafloxacin and levofloxacin powders were commercially obtained (Sigma-Aldrich, St. Louis, MO). Stock solutions of each agent were freshly prepared as single-use aliquots at the beginning of each week and kept frozen at −80°C. aCAMHB was prepared in house by adjusting the pH of CAMHB from 7.3 ± 0.2 to 6.5 ± 0.2 using 1 N hydrochloric acid and a digital pH meter (Seven2Go; Mettler Toledo, Columbus, OH). MICs were determined in triplicate via reference BMD testing according to CLSI guidelines (38). The same 0.5 McFarland standard suspension was used for BMD tests on the same day in CAMHB and aCAMHB concomitantly. Drug concentrations in BMD panels ranged from 0.25 to 16 mg/L for levofloxacin in CAMHB, from 0.5 to 32 mg/L for levofloxacin in aCAMHB and delafloxacin in CAMHB, and from 0.03 to 2 mg/L for delafloxacin in aCAMHB. Low off-scale MICs were reported as observed (e.g., ≤0.25 or ≤0.03 mg/L), and high off-scale MICs (e.g., >32 or >2 mg/L) were converted and reported as the next highest log_2_ dilution (e.g., ≥64 or ≥4 mg/L) (39). Modal MIC values from triplicate BMD tests were recorded and are reported as MIC_50_, MIC_90_, and MIC range values for CAMHB and as aMIC_50_, aMIC_90_, and aMIC range values for aCAMHB. Escherichia coli ATCC 25922 and Pseudomonas aeruginosa ATCC 27853 were used as quality control organisms. Susceptibility interpretations for levofloxacin were based on CLSI breakpoints against S. maltophilia, and those for delafloxacin were based on FDA breakpoints against P. aeruginosa (38, 40).

### Time-kill experiments.

Time-kill experiments were performed in triplicate on the same day against a subset of 5 S. maltophilia isolates selected from the original 37 to provide a range of delafloxacin MIC and aMIC values. Experiments were performed according to CLSI guidelines modified using a final volume of 2 mL in deep-well, non-tissue-treated plates (41). A starting inoculum of ~10^6^ CFU/mL was prepared by suspending 3 or 4 isolated colonies selected from a pure overnight culture in 5 mL of sterile saline and adjusting the suspension to a 0.5 McFarland standard, which was subsequently incubated with agitation to ensure log-phase growth and then diluted 1:100 in CAMHB or aCAMHB. Colony counts were performed to ensure final inoculum densities. Time-kill experiments were performed with delafloxacin and levofloxacin tested alone at their respective *fC*_max_ values in CAMHB and aCAMHB. The *fC*_max_ values utilized corresponded to the maximum labeled intravenous dose of each agent, i.e., 300 mg of delafloxacin (*fC*_max_, 1.5 mg/L) and 750 mg of levofloxacin (*fC*_max_, 6.5 mg/L) (42, 43). Assuming no drug degradation over the 24-h experiment, these *fC*_max_ values should result in minimum *f*AUC values of approximately 36 mg · h/L for delafloxacin and 156 mg · h/L for levofloxacin. These values were then indexed to the MIC/aMIC for each agent and assessed in relation to the established FQ *C*_max_/MIC associated with clinical and microbiologic cure of 12.2 and the applicable *f*AUC/MIC 1-log_10_ CFU/mL reduction targets of 60 for delafloxacin against P. aeruginosa and 55 for levofloxacin against S. maltophilia (14, 44, 45). Growth controls without any antibiotic in CAMHB and aCAMHB were included in each experiment. At the prespecified time points of 0, 2, 4, 6, and 24 h, aliquots of 20 µL were removed from the suspensions and serially diluted in log_10_ dilutions. A 50-µL aliquot was then plated on Mueller-Hinton (MH) agar plates using an automated spiral plater (Don Whitley WASP Touch; Microbiology International, Frederick, MD) and incubated at 35°C for at least 24 h prior to enumeration. Colony counts were performed using an automated colony counter (ProtoCOL 3 Plus; Synbiosis, Frederick, MD). The theoretical lower limit of quantitation was 100 CFU/mL. Time-kill curves were generated by plotting the average log_10_ CFU per milliliter versus time to compare the 24-h killing effects of delafloxacin and levofloxacin in CAMHB and aCAMHB. Bactericidal activity was defined as ≥3-log_10_ CFU/mL reduction at 24 h, compared to the starting inoculum (41).
